# NOX4 Mediates Epithelial Cell Death in Hyperoxic Acute Lung Injury Through Mitochondrial Reactive Oxygen Species

**DOI:** 10.3389/fphar.2022.880878

**Published:** 2022-05-19

**Authors:** Anantha Harijith, Prathima Basa, Alison Ha, Jaya Thomas, Anjum Jafri, Panfeng Fu, Peter M. MacFarlane, Thomas M. Raffay, Viswanathan Natarajan, Tara Sudhadevi

**Affiliations:** ^1^ Department of Pediatrics, School of Medicine, Case Western Reserve University, Cleveland, OH, United States; ^2^ Department of Biochemistry and Molecular Genetics, College of Medicine, University of Illinois at Chicago, Chicago, IL, United States; ^3^ Department of Genetics and Genome Sciences, School of Medicine, Case Western Reserve University, Cleveland, OH, United States; ^4^ Department of Pharmacology and Regenerative Medicine, University of Illinois at Chicago, Chicago, IL, United States; ^5^ Department of Internal Medicine, College of Medicine, University of Illinois at Chicago, Chicago, IL, United States

**Keywords:** NADPH oxidase 4, mitochondrial ROS, alveolar type 2 epithelial cell, apoptosis, hyperoxic acute lung injury

## Abstract

Management of acute respiratory distress involves O_2_ supplementation, which is lifesaving, but causes severe hyperoxic acute lung injury (HALI). NADPH oxidase (NOX) could be a major source of reactive oxygen species (ROS) in hyperoxia (HO). Epithelial cell death is a crucial step in the development of many lung diseases. Alveolar type II (AT2) cells are the metabolically active epithelial cells of alveoli that serve as a source of AT1 cells following lung injury. The aim of this study was to determine the possible role of AT2 epithelial cell NOX4 in epithelial cell death from HALI. Wild type (WT), *Nox4* ^*fl/fl*^ (control), and *Nox4* ^*−/−*^ *Spc-Cre* mice were exposed to room air (NO) or 95% O_2_ (HO) to investigate the structural and functional changes in the lung. C57BL/6J WT animals subjected to HO showed increased expression of lung NOX4 compared to NO. Significant HALI, increased bronchoalveolar lavage cell counts, increased protein levels, elevated proinflammatory cytokines and increased AT2 cell death seen in hyperoxic *Nox4* ^*fl/fl*^ control mice were attenuated in HO-exposed *Nox4* ^*−/−*^ *Spc-Cre* mice. HO-induced expression of NOX4 in MLE cells resulted in increased mitochondrial (mt) superoxide production and cell apoptosis, which was reduced in NOX4 siRNA silenced cells. This study demonstrates a novel role for epithelial cell NOX4 in accelerating lung epithelial cell apoptosis from HALI. Deletion of the *Nox4* gene in AT2 cells or silencing NOX4 in lung epithelial cells protected the lungs from severe HALI with reduced apoptosis and decreased mt ROS production in HO. These results suggest NOX4 as a potential target for the treatment of HALI.

## Introduction

Supplemental oxygen therapy is often inevitable in the treatment of serious lung diseases like COVID-19 pneumonia (Dias-Freitas et al., 2016). However, prolonged exposure of patients with acute respiratory distress syndrome (ARDS) to a high fraction of inspired oxygen (FiO_2_) i.e., hyperoxia (HO) causes severe hyperoxic acute lung injury (HALI). HO overwhelms the antioxidant defense systems of the body leading to oxidative stress, triggering several pathological processes in the lung. This results in severe lung damage with subsequent deterioration of respiratory function (Kallet and Matthay, 2013). Various experimental strategies to reduce increased ROS showed some beneficial roles in animal models; however, were ineffective clinically (Welty-Wolf et al., 1997; Otterbein et al., 1999). Hence, there is an immediate need to identify new targets and understand mechanism(s) of HO-induced ROS generation in different cell types in the lung for the development of potential therapies.

Cellular respiration is the prime source for generation of ROS (Auten and Davis, 2009). NADPH oxidases (NOXs) are also major sources of cellular ROS and have been a focus of extensive research due to their critical function in producing ROS under normal and patho-physiological conditions (Mittal et al., 2014; Panday et al., 2015). Over-activity or increased expression of NOX proteins can lead to oxidative stress causing cellular damage (Tarafdar and Pula, 2018). While NOX1, 2, 3, and 5 generate superoxide, NOX4 produces hydrogen peroxide (H_2_O_2_) (Takac et al., 2011; Nisimoto et al., 2014). Interestingly, NOX4 is localized both in the mitochondrial and cytosolic compartment of mammalian cells (Carnesecchi et al., 2009; Brown and Griendling, 2009; Pendyala et al., 2009) as well as in the nucleus (Anilkumar et al., 2013). NOX2/NOX4 activation by HO has been shown to promote oxidative stress (Parinandi et al., 2003; Chowdhury et al., 2005; Usatyuk et al., 2007; Pendyala et al., 2011) in endothelial cells. Dual Oxidase 2 in mouse lung epithelia was found to be essential for HO-induced acute lung injury (Kim et al., 2014; Dias-Freitas et al., 2016). NOX2 has been extensively studied in lungs and generates high levels of ROS in neutrophils as a central mechanism of host defense against microbial infection (Paiva and Bozza, 2014). NOX2, for its activity, needs assembly of other cytosolic components such as *p47* ^*phox*^, *p67* ^*phox*^ and Rac1 in the plasma membrane (Bernard et al., 2014). In contrast, NOX4 is constitutively active, seems not to require any other component for its activity and generates low levels of H_2_O_2_ under basal conditions; however, not much is known about its role in HALI. NOX4 has recently gained attention as it is now known to regulate several pathological processes including cellular differentiation (Lee et al., 2014), host defense (Kim et al., 2017), and tissue fibrosis (Hecker et al., 2009).

The pathophysiology of HALI suggests AT2 to be one of the most vulnerable lung cell types. AT2 cells are essential for lung regeneration after injury (Olajuyin et al., 2019). It was demonstrated that NOX4 deficiency or acute treatment with a NOX4 inhibitor blunted TGFβ1-induced alveolar epithelial cell death and prevented subsequent pulmonary fibrosis in a murine model of lung fibrosis (Carnesecchi et al., 2011). NOX4 also mediates myofibroblast activation and fibrogenic responses to lung injury (Hecker et al., 2009). There is growing evidence that suggests NOX4 as a potential target for the treatment of ARDS/ALI patients during both the acute and the proliferative stage (Manoury et al., 2005; Bernard et al., 2017). Our data have shown elevated NOX4 expression in whole lung tissue as well as lung epithelial cells upon HO treatment which led to our hypothesis that AT2 NOX4 mediates HALI and AT2 cell specific deletion of *Nox4* will attenuate HO injury.

## Materials and Methods

### Animal Experiments and Procedures

All experimental procedures were approved by the Institutional Animal Care and Use Committee (protocol #2020-0052) of the Case Western Reserve University (CWRU). All animals were treated in accordance with the NIH guidelines for the care and use of laboratory animals. *Nox4* ^*fl/fl*^ mice used in this study were described previously (Matsushima et al., 2013; Fu et al., 2021). To determine the role of AT2 epithelial cell NOX4 in HO-mediated lung inflammatory injury, mice harboring a deletion of *Nox4* in AT2 cells (*Nox4* ^*−/−*^ *Spc-Cre*) were generated. The *Nox4* ^*fl/fl*^ and the *Nox* ^*+/-*^ *Spc-Cre* mice were gifted by Dr. V Natarajan, Dept. of Pharmacology and Regenerative Medicine, University of Illinois at Chicago. Both male and female mice, 8 weeks old, were used for the experiments. Temporal deletion of *Nox4* in *Nox* ^*+/-*^ *Spc-Cre* mice was achieved by intraperitoneal injection of tamoxifen (200 mg/kg dissolved in corn oil) for five consecutive days. The animals were used for experiments 2 weeks later. *Nox4* ^*fl/fl*^ mice that served as controls also received the same dosage and time course of tamoxifen injection and experimentation. The mice were exposed to room air/ normoxia (NO) or 95% hyperoxia (HO) for 66 h. A 66 h timepoint was chosen based on the preliminary data collected at either 48 or 72 h. At 48 h, there was no significant injury observed (data not shown) and at 72 h, the animals appeared seriously morbid. Following exposure to NO or HO, the mice were humanely euthanized to collect lungs for histology, bronchoalveolar lavage (BAL) and other analyses. Euthanasia was by anesthetic overdose (intraperitoneal injection of a 100 mg/kg ketamine and 10 mg/kg xylazine mix) followed by exsanguination. Treatment groups of 6–10 mice (both male and female) were used for each of the experiments and representative data are shown.

### Lung Preparation for Histology

The animals were euthanized, the trachea intubated, and the left lung lobe was inflated with neutral buffered formalin at 20 cm of H_2_O pressure. Following fixation for another 24 h, the lungs were processed for paraffin embedding and sectioning. Lung tissue was cut into 5 μm sections for subsequent alveolar analyses after hematoxylin and eosin (H&E) staining. We have used two serial sections (six fields/section) from each animal and a total of six animals per group. Assessment of acute lung injury was done for infiltration of neutrophils, alveolar wall thickening and deposition of proteinaceous debris (Matute-Bello et al., 2011). Briefly, a scoring system with scores of 0, 1 and 2 was used for each of the three criteria mentioned. An average score of 0 indicated absence of injury, 1–2 indicated mild to moderate injury and two indicated severe injury. Scoring was done for all animals and averaged for a group.

### Immunohistochemistry (IHC)

To assess the evaluation of *Nox4* deletion in AT2 cells, the lung tissue sections from *Nox4* ^*fl/fl*^ and *Nox4* ^*−/−*^ *Spc-Cre* mice (*Nox4* deleted) were pretreated with antigen retrieval buffer (10 mM citrate), followed by blocking nonspecific binding with appropriate serum. Sections were then subjected to immunofluorescence co-staining for NOX4 (1:300; 14347-1-AP; Proteintech, Rosemont, IL, United States) and surfactant protein-C/ SPC (1:50; sc-7705; Santacruz, Dallas, TX, United States) to co-localize NOX4 deletion in AT2 cells. This was followed by incubation with respective secondary antibodies (Alexa Fluor 488 and Alexa Fluor 594 from Invitrogen at 1:500) followed by mounting using Vectashield mounting medium containing 4’,6-diamidino-2-phenylindole/DAPI (Vector Laboratories, Burlingame, CA, United States). Appropriate negative controls were run by omitting the primary antibody to confirm nonspecific staining. Images of the immunostained sections were captured using a fluorescence microscope (Leica Microsystems, Wetzlar, Germany) equipped with a Rolera XR CCD camera (Q-Imaging, Surrey, BC, Canada) mounted on a microscope. ImageJ software was used to analyze the stained lung sections and the intensity of staining was measured per area.

### BAL Collection and Analysis

BAL collection was based on our previous protocol (Ha et al., 2020; Sudhadevi et al., 2021). Briefly, the mice were sacrificed, the trachea were exposed, the tubing were inserted and secured, and the whole lung was lavaged with 1 ml of PBS. Cells in the BAL fluid was counted using a cell counter (Bio-Rad). The total protein concentration in BAL fluid was measured using the BCA protein assay according to the manufacturer’s instructions (#23225; Pierce). For cytokine analysis in the BAL fluid, Luminex technology and reagents (R&D Systems, Minneapolis, MN, United States) were used. Murine TNF-α, IL-6, macrophage inflammatory protein 2 (MIP-2), keratinocyte-derived chemokine (KC), and MIP-1 ∝ were analyzed according to the manufacturer’s instructions.

### Analysis of Cell Death

Terminal deoxynucleotidyl transferase dUTP nick end labeling (TUNEL) assay was used per manufacturer’s instructions (The ApopTag Peroxidase *In Situ* Apoptosis Detection Kit, S7100; Millipore Sigma, Billerica, United States). Sections were counterstained with methyl green. The number of TUNEL positive cells were counted in all areas of the section using a light microscope (×20 magnification) and the average number of TUNEL-positive cells per field per animal were calculated.

### Lung Function Measurements

Mice after exposure to NO or HO were anesthetized intraperitoneally with ketamine (100 mg/kg) and xylazine (10 mg/kg), tracheostomized with a metal 18 G cannula and connected to a flexiVent ventilator (SCIREQ, Montréal, QC, Canada). Baseline measurements were performed using forced-oscillation maneuvers and calculated using the ventilator software (flexiWare 5.1, Version 7.2; SCIREQ). Following a recruitment breath to 30 cm H_2_O, three measurements of respiratory system resistance (Rrs), respiratory system elastance (Ers), and dynamic compliance (Crs) were performed (*Snapshot 150*), as well as lung tissue impedance parameters of tissue resistance (G) and tissue elastance (H) (*Quick Prime-3*). Mean values of Rrs, Ers, Crs, G and H for each animal are reported.

### Exposure of Cells to Hyperoxia *In Vitro*

Mouse lung epithelial cells (MLE-12) cultured in DMEM-F12 medium containing 10% FBS and antibiotic (Sigma) were used for the *in vitro* experiments. The cells (∼80% confluence) were exposed to HO (95% O_2_) as described before (Harijith et al., 2016) using a humidity-controlled airtight Billups- Rothenberg modulator incubator chamber (Billups-Rothenberg, CA, United States), flushed with 95% O_2_:5% CO_2_ gas. The concentration of O_2_ inside was continuously monitored using a digital oxygen monitor. The buffering capacity of the cell culture medium was maintained at a pH ∼7.4 during the period of HO exposure.

### Immunoblotting

Protein expression was detected in the mouse lungs and MLE-12 cells by immunoblotting as described earlier (Harijith et al., 2013; Bandela et al., 2021). Lung tissues were pulverized (Bessman tissue pulverizer, # 189475; Spectrum Laboratories Inc.) using a pulverizer, sonicated in RIPA buffer (Sigma, in the ratio 10 volumes of lysis buffer to 1 part by weight of lung tissue) and centrifuged to collect the supernatant. Similarly, cells exposed to HO were lysed in RIPA buffer, sonicated and the supernatants were subjected to protein quantification using the BCA protein assay (Pierce Chemical, Rockford, IL, United States). Samples (20 µg) were subjected to SDS-gel electrophoresis (ThermoFisher, DE, United States) and immunoblotting (#03500216; Hoefer, Fisher Scientific). The following antibodies were used: NOX4 antibody (1:300; 14347-1-AP, Proteintech, IL, United States), β -actin (A15441, Sigma Aldrich, MO, United States) and GAPDH (10494-1-AP, Proteintech, IL, United States). The bands were detected using Pierce ECL (ThermoFisher, DE, United States) or Amersham ECL Prime (Cytiva) followed by exposure to light sensitive Hyperfilm (Amersham Biosciences, Little Chalfont, United Kingdom). ImageJ software (NIH, Bethesda, MD, United States) was used to quantify the relative intensities of protein bands. Results were expressed as a ratio of specific protein signal to the house keeping protein.

### Transient Transfection of Mouse Lung Epithelial Cells

MLE-12 cells at ∼50% confluence were transfected with scRNA (scrambled) or siRNA specific for NOX4 (sc-41587; Santacruz, CA, United States) using Gene Silencer transfection reagent (T500750; Gelantis, San Diego, CA, United States) in serum-free DMEM medium (Sigma) according to manufacturer’s recommendations. After 3 h of transfection, the serum-free medium was replaced with complete MLE-12 medium containing 10% FBS. The cells were cultured for an additional 48h/72 h prior to experiments.

### Treatment of Cells With MitoTEMPO, a Mitochondrial Superoxide Scavenger

MLE-12 cells were pretreated with 10 µM  μ MitoTEMPO (Santacruz, TX, United States) or vehicle control for 2 h and subjected to NO or HO for 24 h and then assessed for mitochondrial superoxide and apoptosis.

### Determination of Mitochondrial ROS

Mitochondrial superoxide generation in MLE1-12 cells upon HO exposure and NOX4 silencing was determined using the MitoSOX Red mitochondrial superoxide indicator (Invitrogen, Greenville, NC, United States), as per the manufacturer’s protocol. The cells were loaded with 5 µM MitoSOX reagent for 15 min. The washed cells were subjected to live cell imaging using a confocal microscope (Leica HyVolution SP8) at ×63 magnification.

### Measurement of Hydrogen Peroxide

MLE-12 cells were cultured in phenol red free medium and then exposed to specific treatments. The cell culture supernatants were collected and H_2_O_2_ concentration was measured using Amplex Red Hydrogen Peroxide/Peroxidase kit (Invitrogen, United States), according to the manufacturer’s instruction.

### Determination of Apoptosis by Flow Cytometry

Flow cytometry was used to assess apoptosis. Mouse lung epithelial cells subjected to various treatments were stained with Annexin V and 7-AAD (PE Annexin V apoptosis kit, #559763; BD, United States) according to the manufacturer’s instructions. Data was analyzed using a BD LSR II (BD Biosciences, United States) flow cytometer. Gating was set based on unstained and single-stained populations.

### Statistical Analysis

All data are expressed as mean ± SEM from a minimum of three independent experiments. GraphPad Prism 9 software was used for statistical analysis. Student’s t-test or two-way ANOVA (Tukey’s *post hoc* tests) were used for comparison of two or more groups respectively. The level of significance was set to ****p < 0.0001, ***p < 0.001, **p <0.01, and *p < 0.05.

## Results

### HO Induces Expression of NOX4 in Lung Tissues

**FIGURE 1:**
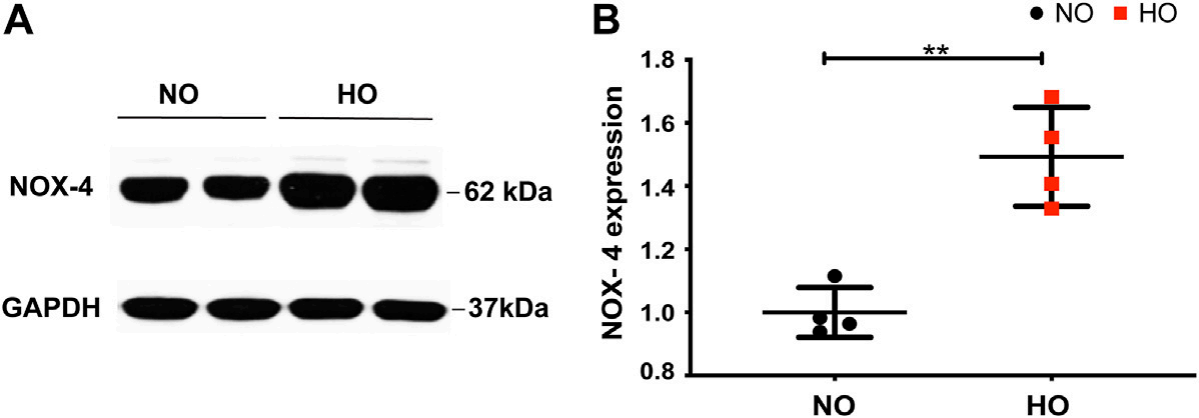
HO induces NOX4 expression in lung tissue. To determine the role of NOX4 in HALI, wild type (WT) mice (C57BL/6J male, ∼25 g body weight) were exposed to either NO or HO (95% oxygen) for 66 h. Total lung tissue protein was then subjected to SDS-PAGE followed by immunoblotting with anti-NOX4 antibody. **(A)** HO significantly enhanced the expression of NOX4 as compared to NO **(B)** Densitometric quantification of NOX4. The data were normalized to GAPDH. *n* = 4. **p < 0.01.

Exposure of wild-type (WT) mice to HO (95% O_2_) for 66 h stimulated NOX4 protein expression in the lungs (∼1.5-fold increase compared to normoxia, NO) (Figures 1A,B).

### Genetic Deletion of *Nox4* in AT2 Cells Protects Mice From HALI

**FIGURE 2:**
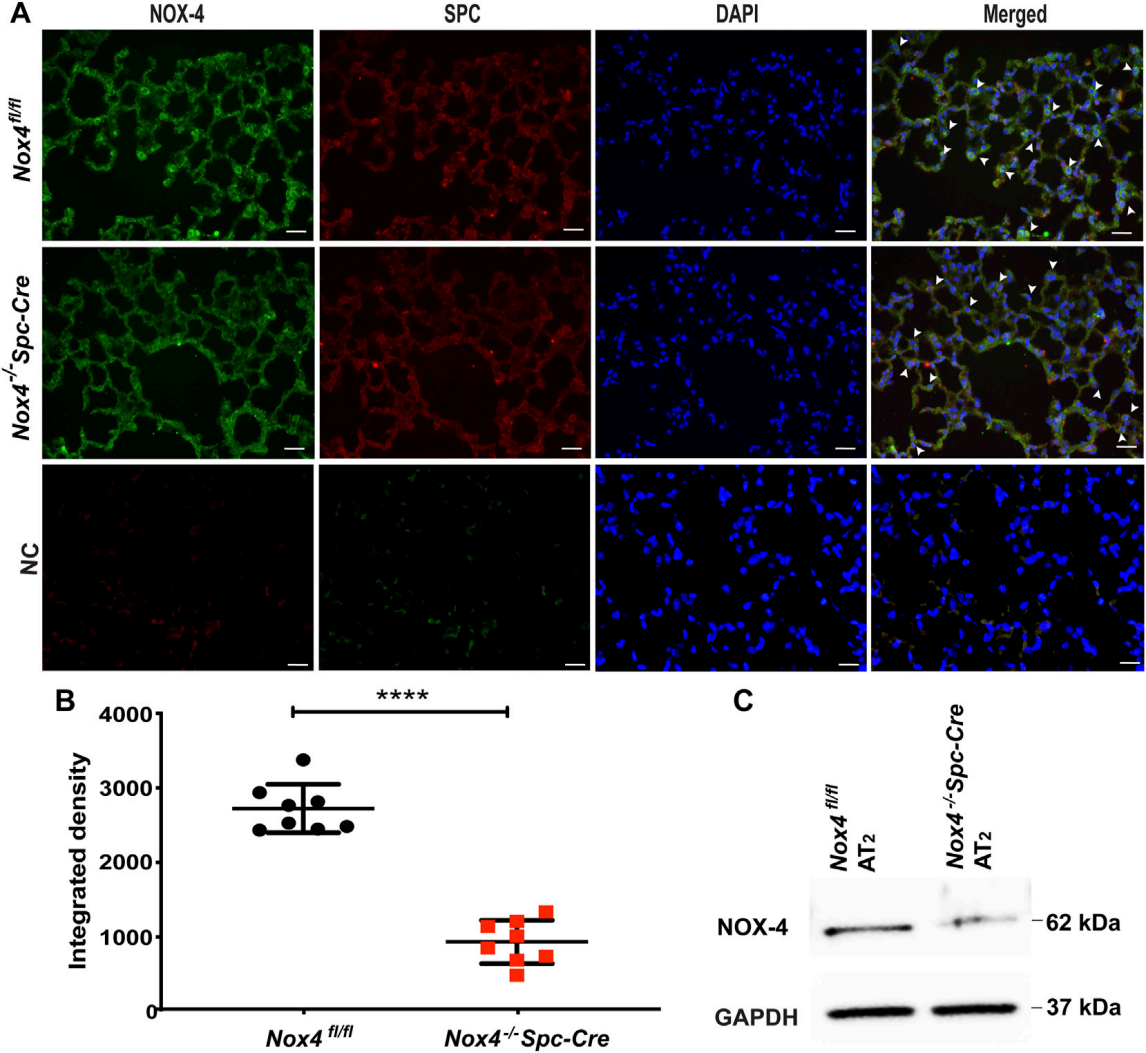
NOX4 expression is downregulated in the lungs and isolated AT2 cells from *Nox4* ^*−/−*^ *Spc-Cre* mice. *Nox* ^*+/-*^ *Spc-Cre* animals were injected with tamoxifen to generate *Nox4* ^*−/−*^ *Spc-Cre* animals and IHC was performed on the harvested lungs. **(A)** Representative immunofluorescent micrographs of lung tissue showing individual staining as well as co-staining of NOX4 and AT2 pneumocyte marker SPC (merge of green and red). Co-localization of NOX4 (green) and SPC (red) could be seen in lungs of *Nox4* ^*fl/fl*^ mice, but not in *Nox4* ^*−/−*^ *Spc-Cre* mice as indicated with white arrows **(B)** Quantified data of NOX4 fluorescence intensity from NOX4-SPC co-stained lung tissue **(C)** Expression of NOX4 in primary AT2 cells isolated from *Nox4* ^*fl/fl*^ and *Nox4* ^*−/−*^ *Spc-Cre* mice lung. *n* = 3 ****p < 0.0001.

*Nox4* ^*−/−*^ *Spc-Cre* animals were generated and validated. Immunofluorescence staining of lung tissue sections demonstrated co-localization of NOX4 (green) with SPC (red) in the lung alveolar epithelial cells of *Nox4* ^*fl/fl*^ mice but not in the tamoxifen induced *Nox4* ^*−/−*^ *Spc-Cre* mice (Figure 2A), indicating a significant knockdown of *Nox4* as quantified by ImageJ analysis (2722 AU for *Nox4* ^*fl/fl*^ lung tissue as compared to 932 AU for *Nox4* ^*−/−*^ *Spc-Cre*) (Figure 2B). Western blot of lysates with AT2 cells isolated from *Nox4* ^*−/−*^ *Spc-Cre* animals showed significant NOX4 knockdown compared to *Nox4* ^*fl/fl*^ (Figure 2C).

**FIGURE 3:**
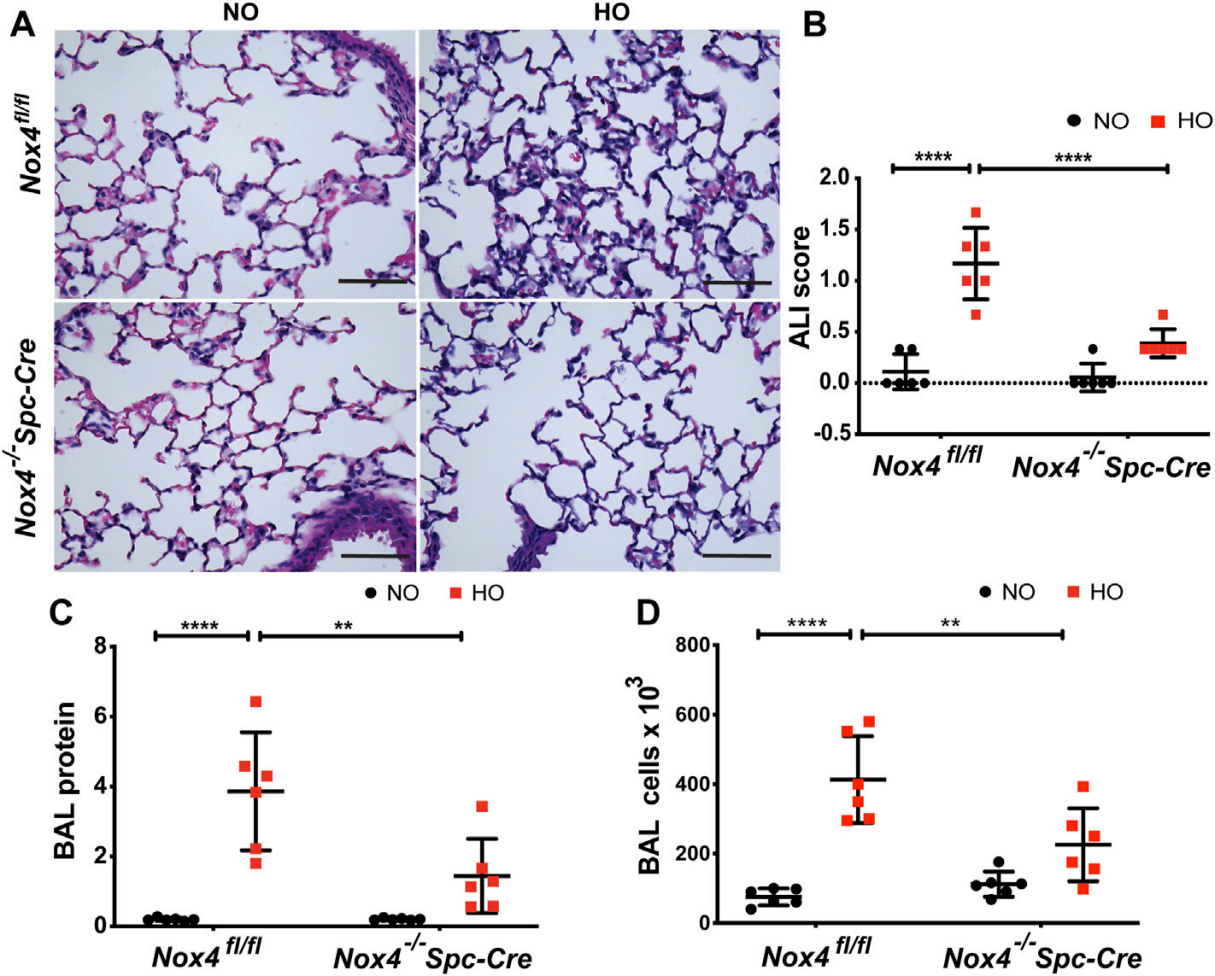
Genetic Deletion of *Nox4* in AT2 cells of mice reduced HALI. *Nox4* ^*fl/fl*^ control mice and *Nox4* ^*−/−*^ *Spc-Cre* mice induced with tamoxifen and were subjected to NO or HO for 66 h. **(A)** Representative H&E photomicrographs of lung tissue sections from *Nox4* ^*fl/fl*^ and *Nox4* ^*−/−*^ *Spc-Cre* mice exposed to NO or HO. Quantitative data of **(B)** ALI scores based on the infiltrating neutrophils, alveolar wall thickening and proteinaceous debris deposition **(C)** BAL protein and **(D)** cell counts were elevated in HO exposed control mice and were attenuated by epithelial deletion of *Nox4*. Scale bar = 100 µm. *n* = 6. ****p < 0.0001, ***p < 0.001, **p < 0.01 and *p < 0.05.

**FIGURE 4:**
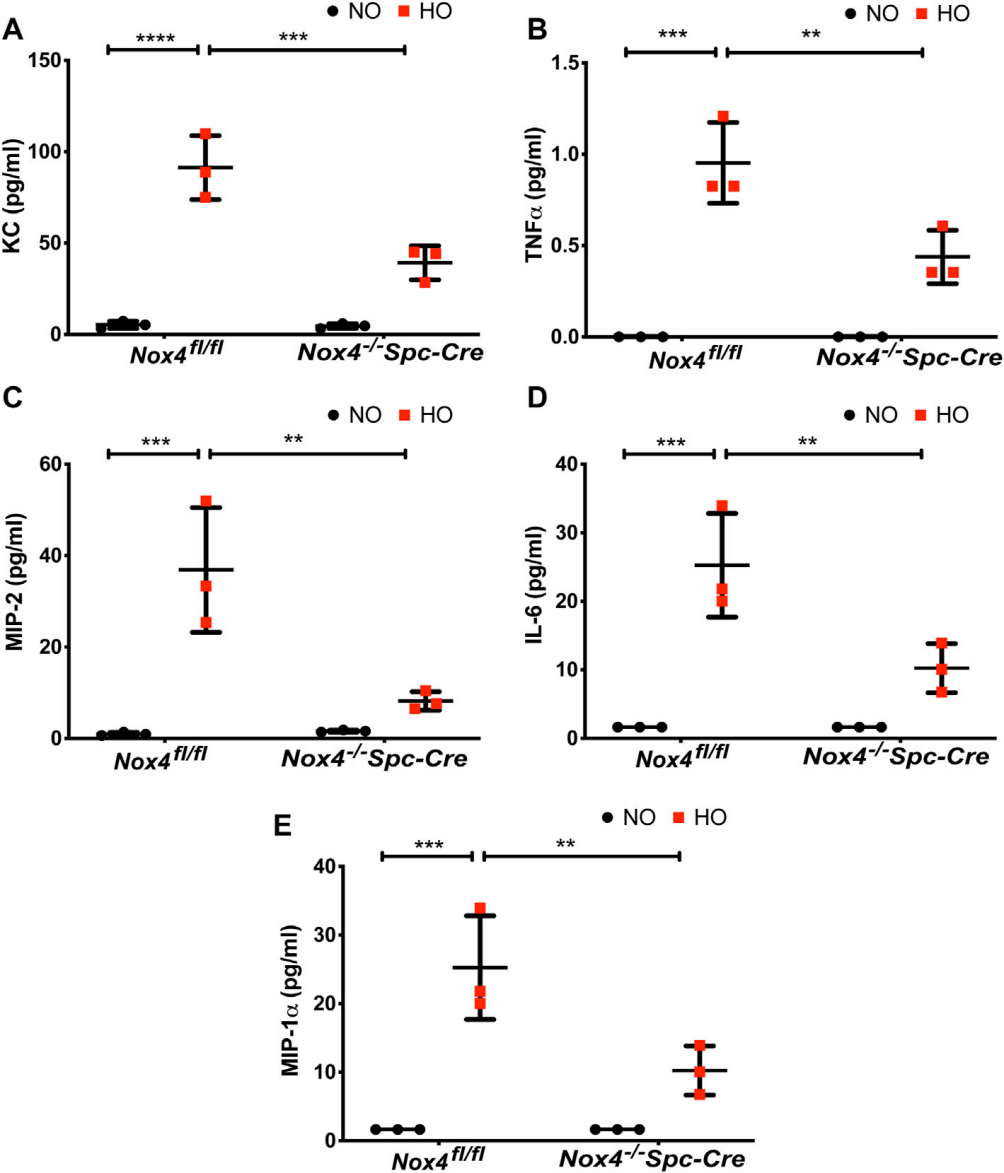
Nox4 deletion in AT2 cells of mice reduced inflammatory cytokine profile. *Nox4* ^*fl/fl*^ control mice and *Nox4* ^*−/−*^ *Spc-Cre* mice were subjected to NO or HO for 66 h.The BAL fluid collected were subjected to multiplex assays to determine the cytokine profiles. **(A)** KC, **(B)** TNF- ∝ , **(C)** MIP-2, **(D)** IL-6 and **(E)** MIP-1 ∝ showed a significant elevation in the BAL fluid of control HO group which were attenuated by epithelial deletion of *Nox4*, expressed as pg/ml. *n* = 3. ****p < 0.0001, ***p < 0.001 and **p < 0.01.

The role of NOX4 in HALI was investigated by exposing *Nox4* ^*fl/fl*^ and *Nox4* ^*−/−*^ *Spc-Cre* mice to NO or HO. As shown in Figure 3A, H & E staining of lung tissues harvested from *Nox4* ^*fl/fl*^ exposed to HO demonstrated significant HALI as determined by increased infiltration of neutrophils, alveolar wall thickening and presence of proteinaceous debris in the alveolar space. ATS scoring was used for quantification of lung injury and showed significantly less injury in the hyperoxic *Nox4* ^*−/−*^ *Spc-Cre* mice (0.38 which is 3.5 fold compared to HO) (Figure 3B) compared to HO (1.16 which is ∼10 fold compared to NO). Increased cell count, as well as protein levels in BAL fluid, were also observed with HO exposure (Figures 3C,D). These responses were noted to be significantly lower in the *Nox4* ^*−/−*^ *Spc-Cre* mice. A significant elevation of the inflammatory cytokines (Figure 4), **(A)** KC (∼91 pg/ml in HO against ∼5 pg/ml in NO), **(B)** TNF- ∝ (∼0.9 pg/ml in HO against ∼0.1 pg/ml in NO), **(C)** MIP-2 (∼36 pg/ml in HO against ∼1 pg/ml in NO), **(D)** IL-6 (∼25 pg/ml in HO against ∼2 pg/ml in NO) and **(E)** MIP-1 ∝ (∼11 pg/ml in HO against ∼0.3 pg/ml in NO) were observed in the BAL fluid from HO exposed *Nox4* ^*fl/fl*^ mice. This increase was attenuated significantly for KC (∼40 pg/ml), TNF- ∝ (∼0.4 pg/ml), MIP-2 (∼8 pg/ml), IL-6 (∼10 pg/ml) and MIP-1 ∝  (∼3 pg/ml) as compared to HO *Nox4* ^*fl/fl*^ controls in the *Nox4* ^*−/−*^ *Spc-Cre* mice.

### Genetic Deletion of *Nox4* Reduced Epithelial Cell Death Induced by HALI

**FIGURE 5:**
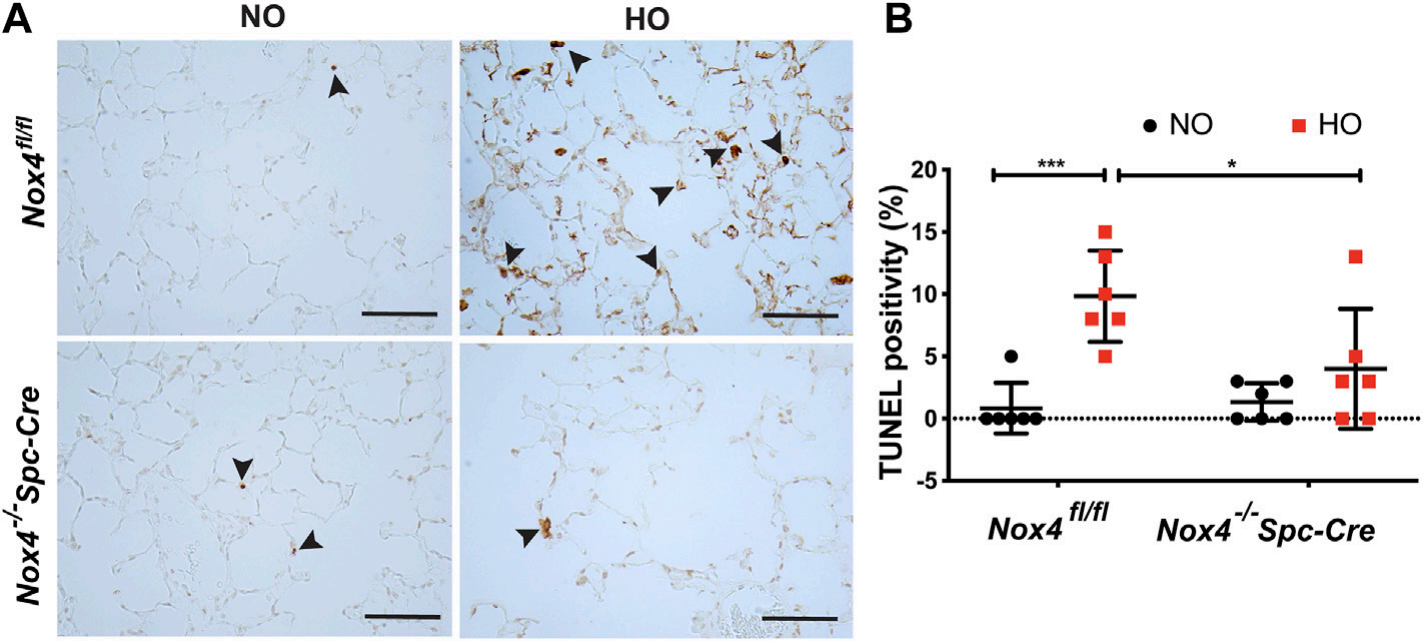
Deletion of *Nox4* reduced AT2 cell death induced by HALI. **(A)** Representative immunohistochemical TUNEL assay micrographs of lung tissues from *Nox4* ^*fl/fl*^ and *Nox4* ^*−/−*^ *Spc-Cre* mice exposed to NO or HO **(B)** The quantified data indicated a lower percentage of TUNEL-positive apoptotic cells in the hyperoxic *Nox4* ^*−/−*^ *Spc-Cre* group. *n* = 6. ***p < 0.001, **p < 0.01 and *p < 0.05.

Next, we investigated epithelial cell apoptosis in HALI. TUNEL staining analysis in *Nox4* ^*fl/fl*^ HO group showed a massive increase in the percentage of TUNEL-positive cells (∼10% in HO as compared to ∼1% in NO) indicating increased AT2 cell death. In contrast, no significant increase in TUNEL-positive cells (∼4% as compared to HO) was observed in *Nox4* ^*−/−*^ *Spc-Cre* HO mice (Figure 5A). The differences between the HO control and the HO *Nox4* ^*−/−*^ *Spc-Cre* mice were statistically significant (Figure 5B).

### Genetic Deletion of *Nox4* in AT2 Cells Prevents Lung Dysfunction Induced by HALI

**FIGURE 6:**
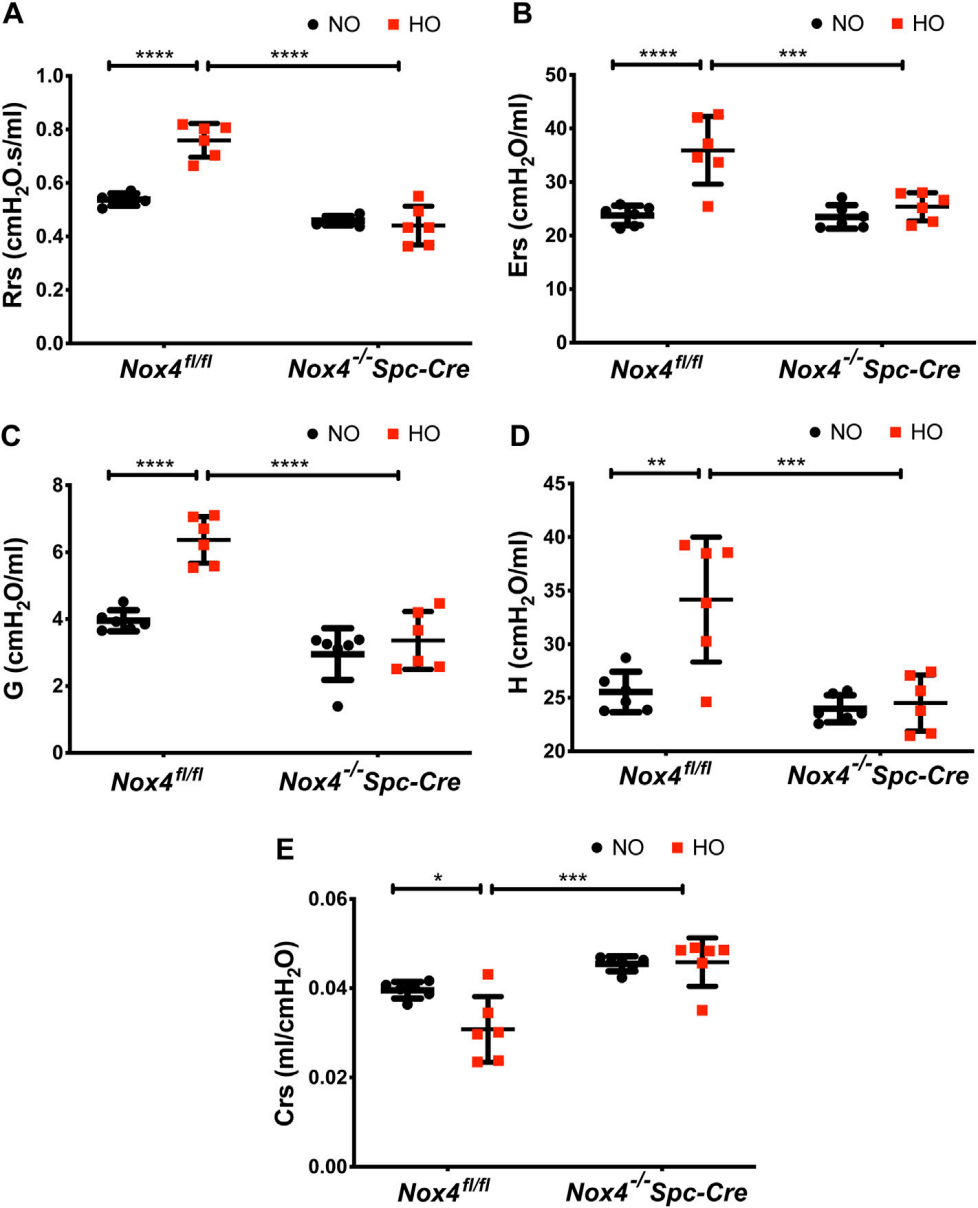
Deletion of *Nox4* in AT2 cells prevented the sustained lung injury induced by HO. HO caused an increase in baseline **(A)** respiratory system resistance (Rrs), **(B)** respiratory system elastance (Ers), **(C)** tissue dampening/tissue resistance (G), **(D)** tissue elasticity (H) and a decrease in **(E)** dynamic compliance (Crs) following HO in control *Nox4* ^*fl/fl*^ mice as measured by forced oscillatory maneuvers on a flexiVent commercial ventilator. These responses were reduced upon knocking out *Nox4*. n = 6. ****p < 0.0001, ***p < 0.001, **p < 0.01 and *p < 0.05.

Hyperoxia resulted in higher baseline respiratory system resistance (Rrs: 0.75 cmH_2_O.s/ml) and respiratory system elastance (Ers: 35.93 cmH_2_O/ml) when compared to NO controls. These were reduced significantly to 0.44 cmH_2_O.s/ml and 25.38 cmH_2_O/ml in hyperoxic animals by genetic deletion of *Nox4* (Figures 6A,B) as compared to control HO group. We also observed significant changes in lung tissue impedance parameters, such as tissue resistance/tissue dampening (G) and tissue elastance (H). The G increased from 3.27 to 6.4 cmH_2_O/ml which is reduced to 3.36 cmH_2_O/ml in the *Nox4* ^*−/−*^ *Spc-Cre* group. H increased from 25 to 34 cmH_2_O/ml but remained low in the *Nox4* deleted group (∼24 cmH_2_O/ml) (Figures 6C,D). Compliance is the inverse of elastance, and we noted a decrease in Crs (∼0.03 cmH_2_O/ml) in the HO group which was significantly increased to ∼0.04 cmH_2_O/ml in the *Nox4* ^*−/−*^ *Spc-Cre* group (Figure 6E).

### HO Induces NOX4 Expression in Lung Epithelial Cells

**FIGURE 7:**
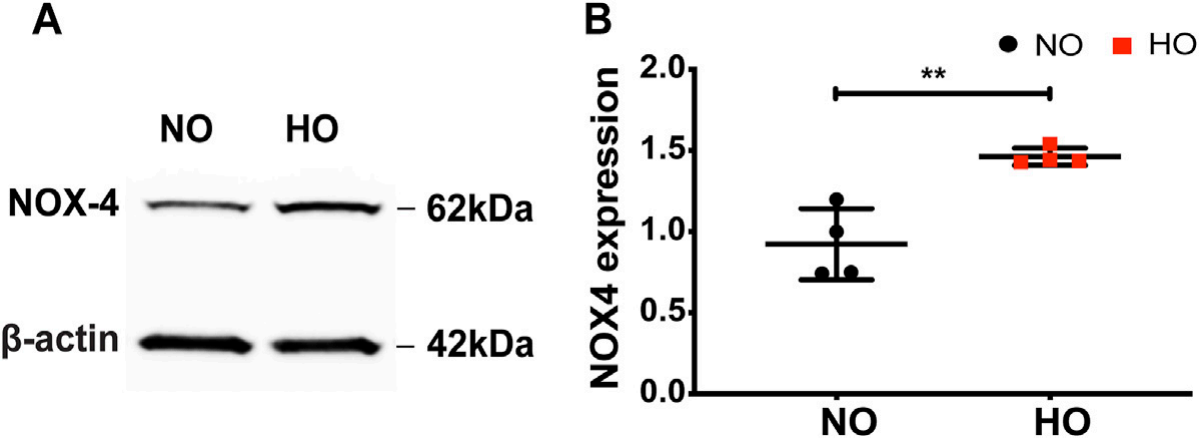
HO induces NOX4 expression in lung epithelial cells. **(A)** MLE-12 cells were exposed to NO or HO and total cell lysates were subjected to Western blotting with anti-NOX4 antibody. Increased expression of NOX4 was observed in the HO group compared to NO **(B)** Densitometric quantification of NOX4. The data were normalized to GAPDH. *n* = 4. **p < 0.01 and *p < 0.05.

Proteins extracted from primary MLE-12 cells exposed to HO showed increased expression of NOX4. Quantitative analysis by ImageJ showed significant increase in NOX4 expression (∼1.5 fold) (Figures 7A,B).

### Inhibition of NOX4 Activity Attenuates HO-Induced Mitochondrial Superoxide and Hydrogen Peroxide Production in Lung Epithelial Cells

**FIGURE 8:**
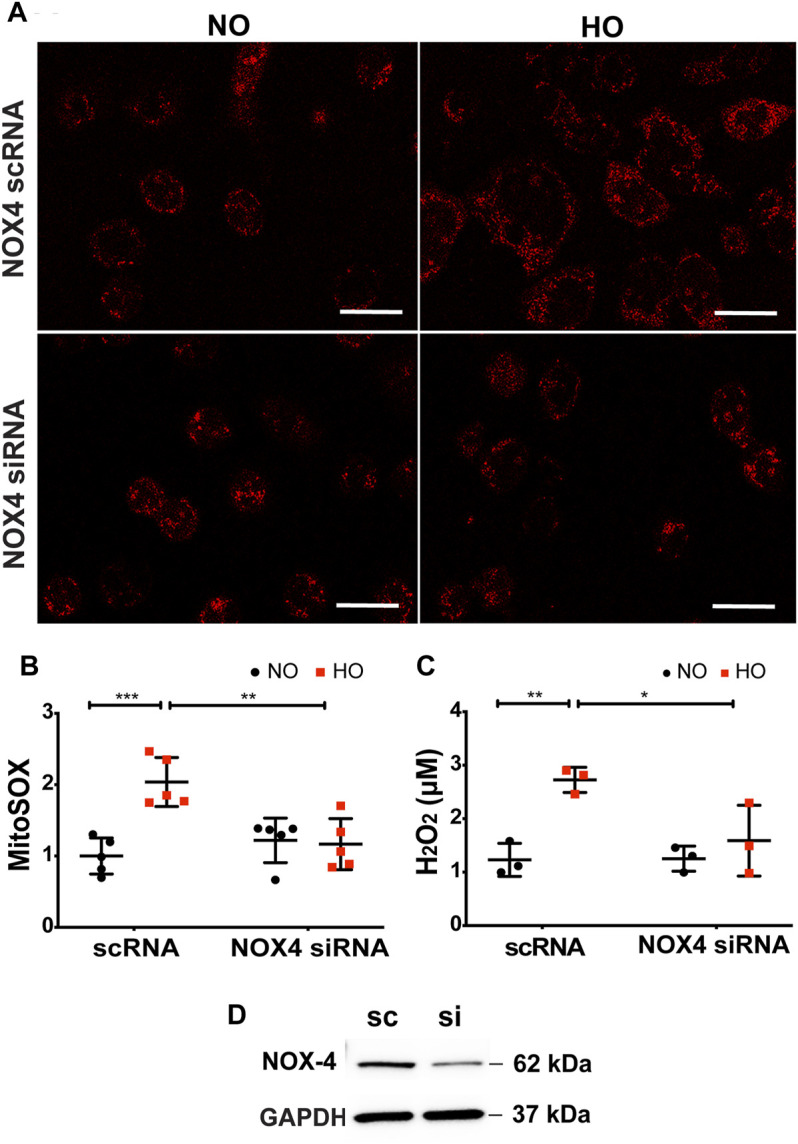
Silencing NOX4 reduces HO-induced increase in mt ROS production and apoptosis. **(A)** Representative confocal images of scRNA and NOX4 siRNA treated MLE-12 cells exposed to NO or HO and stained with MitoSOX **(B)** Quantitative data of mt ROS intensity measured as arbitrary units showed a decrease in HO-induced mt ROS with inhibition of NOX4 activity **(C)** Amplex red H_2_O_2_ assay of the cell culture supernatant showed increased release of H_2_O_2_ from MLE-12 cells exposed to HO compared to NOX4 siRNA treated cell supernatant **(D)** Immunoblots for NOX4 protein showed a significant reduction in the siRNA treated cells relative to control scRNA cells confirming efficient transfection. *n* = 3. ***p < 0.001, **p < 0.01, *p < 0.05.

The role of NOX4 in HO-induced mt ROS was investigated. HO considerably increased MitoSOX fluorescence intensity (∼2 fold more as compared to NO) as well as H_2_O_2_ release (∼2.7 µM as compared to 1.2 µM for NO). Silencing NOX4 in MLE-12 cells using NOX4 siRNA for 24 h significantly attenuated HO-induced mitochondrial superoxide production (∼1.2 fold as compared to HO) as determined by MitoSOX (Figures 8A,B) as well as H_2_O_2_ release (∼1.6 µM as compared to HO) determined by the Amplex red assay (Figure 8C). Western blot of MLE-12 cells confirmed NOX4 knockdown in the siRNA treated group compared to scRNA (Figure 8D). These results show a role for NOX4 in HO-mediated mt ROS production in lung epithelial cells.

### Effect of NOX4 Silencing on Lung Epithelial Cell Apoptosis

**FIGURE 9:**
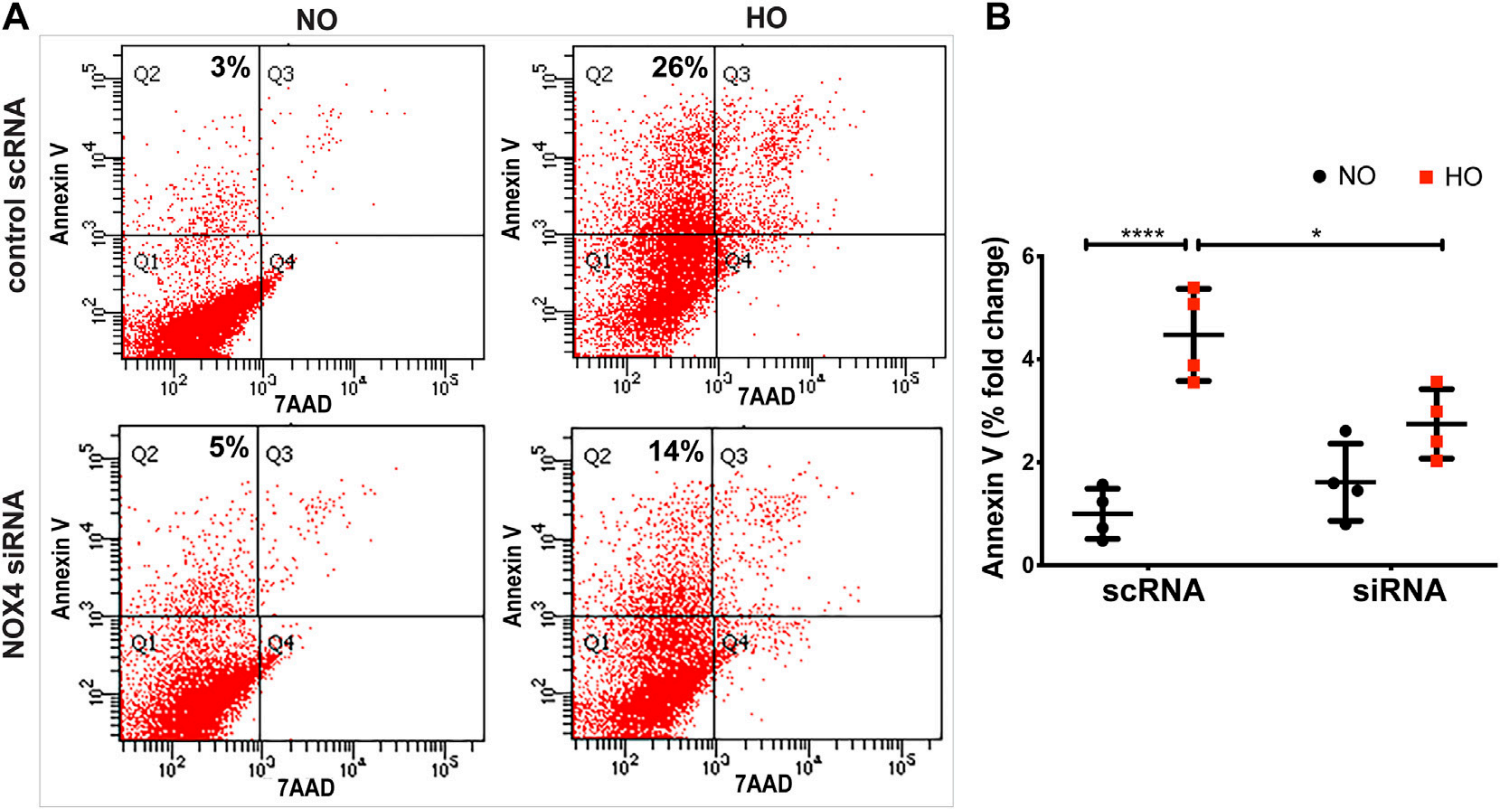
NOX4 expression regulates HO-induced apoptosis in lung epithelial cells. MLE-12 cells were transfected with NOX4 siRNA or control scrambled RNA for 24 h before HO exposure and apoptosis was assessed by flow cytometry. **(A)** Representative dot plots of cells stained with Annexin and 7-AAD **(B)** Graphs representing the percent fold-change of Annexin V- positive cells showing an increase in percentage fold change of apoptosis in HO group which was reduced in the siRNA treated cells. *n* = 3. ***p < 0.001, *p < 0.05.

Exposure of MLE-12 cells to HO for 48 h significantly increased the percentage of Annexin V positive early apoptotic cells and this increase was attenuated with NOX4 silencing (Figures 9A,B) suggesting a pathological role for NOX4 in promoting apoptosis. The average percentage fold change was ∼4.5 for HO as compared to NO and this was reduced to ∼2.5 for NOX4 silenced cells.

### Effect of MitoTEMPO on HO-Induced Lung Epithelial Cell mt ROS Production and Apoptosis

**FIGURE 10:**
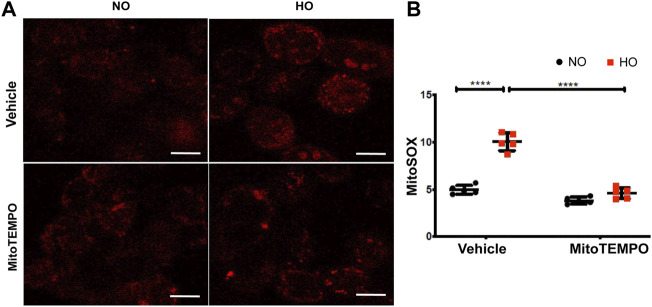
HO-induced ROS generation in MLE-12 cells is attenuated by MitoTEMPO. MLE-12 cells were pretreated with vehicle or MitoTEMPO (10 µm) for 1 h and challenged with HO for 48 h. Mitochondrial superoxide production was assayed by MitoSOX red reagent (5 µm) **(A)** Representative images of MLE-12 cells stained with MitoSOX and **(B)** Quantified fluorescence intensity measured as arbitrary units showed an increase in MitoSOX staining with HO and decrease with MitoTEMPO treatment. *n* = 3. ****p < 0.0001.

**FIGURE 11:**
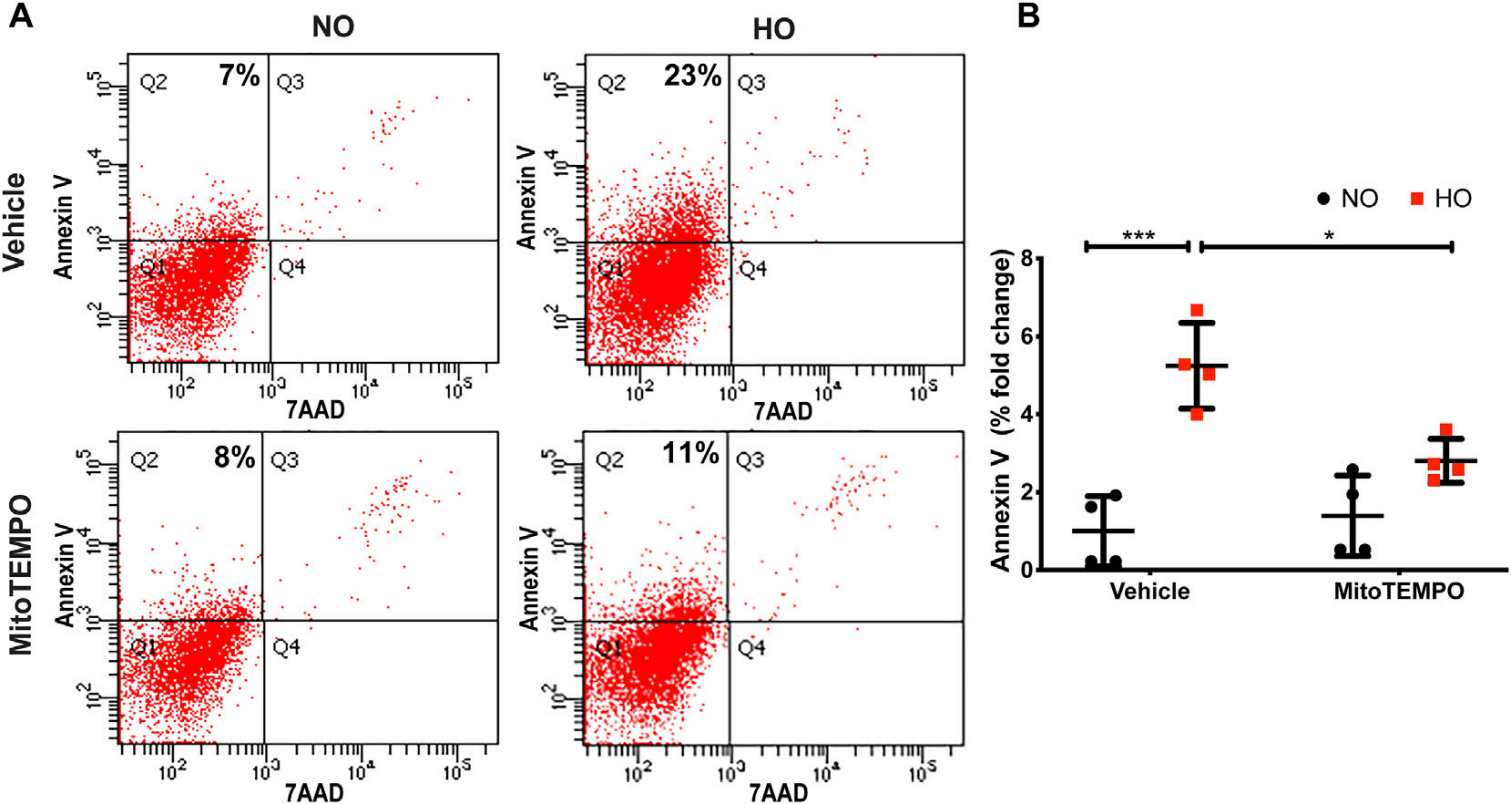
MitoTEMPO attenuates HO-induced apoptosis in MLE-12 cells. MLE-12 cells were pretreated with 10uM MitoTEMPO for 1 h followed by HO for 48 h. The cells were stained with Annexin V and 7-AAD and were subjected to flow cytometry. **(A)** Representative dot plots of cells after staining with Annexin V and 7-AAD **(B)** Quantification of the percentage of Annexin V- positive apoptotic cells which is expressed as fold-change showing increased apoptotic cells in HO which is significantly reduced with MitoTEMPO treatment. *n* = 4. ***p < 0.001, *p < 0.05.

HO induced an increase in mt ROS production as determined by MitoSOX (∼10 AU in HO as compared to ∼5 AU for NO). Scavenging mitochondrial superoxide with MitoTEMPO reduced this HO-induced mitochondrial superoxide production to ∼4.5 AU (Figure 10). Also, the HO-induced increase in the percentage of epithelial cell apoptosis (∼5% fold change compared to NO) was attenuated (∼2.5 fold as compared to HO) by pretreatment with MitoTEMPO (Figure 11).

## Discussion

Prolonged exposure to hyperoxia (HO) can result in lung tissue damage, which can lead to the development of both acute and chronic lung injury (Northway et al., 1967; Wispe and Roberts, 1987). Despite recent advances, the precise molecular mechanisms by which HO induces lung injury remain unresolved and was the subject of this study. We observed an increase in the expression of NOX4 in WT mice exposed to HO and genetic deletion of *Nox4* in lung AT2 epithelial cells (*Nox4* ^*−/−*^ *Spc-Cre)* conferred protection against HO-induced HALI.

Reactive oxygen species (ROS) play a significant role in HALI and induces death of different cell types (Buccellato et al., 2004; Bhandari, 2008; Attaye et al., 2017). The pathophysiology of HALI begins with oxidative stress induced destruction of alveolar-capillary barriers followed by an extensive inflammatory response augmented by chemotactic factors (Slutsky, 1999; Bhandari and Elias, 2006). Further, endothelial barrier disruption is followed by death of the Type I pneumocytes. The Type II pneumocytes are more tolerant to HO, though evidence of DNA damage to these cells has been reported (Roper et al., 2004). As several lung cell types are involved in the process of ROS generation, it is imperative to understand the involvement of specific type of cells as well as subcellular organelles in this process. In this study, using cell-specific knockdown of *Nox4* in the AT2 cells of the mouse lung, we have defined a novel role for mitochondrial NOX4 in lung AT2 epithelial cell in ROS generation leading to lung epithelial cell apoptosis.

The NOX family, comprising of NOX1–5, are differentially expressed in mammalian cells (Bedard and Krause, 2007; Pendyala and Natarajan, 2010). ROS generation by NADPH oxidases has a context-dependent effect. It has a remarkable role in host defense against invading pathogens in addition to established roles in cellular signaling (Bernard et al., 2017), cell growth (Reddy et al., 2011), cell differentiation and survival (Cucoranu et al., 2005; Hecker et al., 2009). It also contributes to cell death and or cellular senescence (Geiszt et al., 2000). NOX enzymes are involved in the development of various lung diseases. Lung injury induces oxidative stress *via* NOX proteins (Sadikot et al., 2006; Fu et al., 2013). Lung epithelial cells express NOX2 and NOX4, of which NOX4 is involved in epithelial apoptosis (Carnesecchi et al., 2011). It has been demonstrated in NOX4-deficient mice that ROS generation by NOX4 is crucial for the induction of alveolar epithelial cell death and subsequent development of lung fibrosis (Carnesecchi et al., 2011). It is well known that in viral infections, NOX4 upregulation might be part of the response to the infection leading to protective apoptosis of the infected cell (Grandvaux et al., 2007). However, the role of AT2 NOX4 in regulating HALI is unclear. In this study, we have demonstrated a critical role for AT2 specific NOX4 in promoting oxidative stress and lung injury induced by HO, as AT2 specific knockdown of *Nox4* showed protection against pulmonary injury. We found elevated levels of NOX4 in the lungs of HO exposed mice as well as the lung epithelial cells exposed to HO. He et al. showed that NOX4 was elevated in lung macrophages from subjects with asbestosis, and mice harboring a deletion of NOX4 in lung macrophages were protected from asbestos-induced fibrosis (He et al., 2019).

HALI is characterized by an extensive inflammatory response and impaired gas exchange leading to pulmonary edema, respiratory failure and death (Klekamp et al., 1999; Slutsky, 1999). In our study, we noted HALI evidenced by a significant increase in infiltrating neutrophils, alveolar wall thickening and proteinaceous debris deposition in the HO group which was reduced with *Nox4* deletion. Elevated BAL proteins, cell counts, and cytokines were observed in HO control mice which was reduced in mice with AT2 specific genetic deletion of *Nox4*. A similar protection from Pseudomonas aeruginosa induced lung injury was observed upon deletion of *Nox4* in lung epithelium. *Nox4* deletion reduced ROS generation, histone acetylation, and inflammation induced by the bacteria (Fu et al., 2021). Exposure to HO in *Nox4* ^*fl/fl*^ mice resulted in significantly higher Rrs, Ers, G, and H with a decreased Crs when compared to NO controls (Figure 6). Conversely, lung mechanics measured in *Nox4* ^*−/−*^ *Spc-Cre* mice exposure to HO did not significantly differ from NO *Nox4* ^*−/−*^ *Spc-Cre* mice. We also observed significant changes in lung mechanic measurements between the HO exposed *Nox4* ^*fl/fl*^ mice and HO exposed *Nox4* ^*−/−*^ *Spc-Cre* mice, indicating protection from HALI lung function changes when *Nox4* was selectively deleted from the AT2 cells.

Studies indicate that multimodal cell death occurs in lung epithelial cells upon exposure to prolonged HO (Kazzaz et al., 1996; Jyonouchi et al., 1998; Mantell et al., 2002). There are several methods to assess epithelial cell apoptosis (Waxman et al., 1998, 11; Buckley et al., 1998; Mantell et al., 2002; Wang et al., 2003; Zhang et al., 2003; Zhang and Foda, 2004). Our study, based on TUNEL assay reports, showed increased apoptosis of AT2 cells in the HO exposed mouse lungs which was reduced with *Nox4* deletion. It has been noted that AT2 epithelial cells isolated from HO-exposed rat lungs and cultured cells exhibited apoptotic characteristics (Buckley et al., 1998). Complementary to our results, NOX4 deficiency was demonstrated to decrease bleomycin-induced death of alveolar epithelial cells (Carnesecchi et al., 2011). In another study, ROS released from fibroblasts led to epithelial cell death in pulmonary fibrosis (Waghray et al., 2005). Our results suggest that AT2 NOX4 induced mt ROS and caused epithelial cell death.

We noted that NOX4, under HO, augmented mt ROS production leading to epithelial cell apoptosis. ROS outlines diverse oxidant molecules possessing distinctive biological functions and properties and biological functions (Sies et al., 2022). We focused on H_2_O_2_ and O_2_^.-^. MitoSOX was used to measure mitochondrial superoxide and amplex red assay the H_2_O_2_ levels. The HO-induced surge in mt ROS (superoxide and H_2_O_2_) was reduced upon down-regulation of *Nox4* with Nox4 siRNA treatment and scavenging of mt ROS by MitoTEMPO (Figures 8, 9). Eventhough these methods are widely used, there are concerns regarding the accuracy of these methods in measuring intracellular ROS (Kalyanaraman, 2020). Novel genetically encoded fluorescent H_2_O_2_ probes and superoxide sensors are available to study intracellular ROS (Kalyanaraman, 2020; Mailloux, 2021). NOX4 has been shown to induce mt ROS and modulate mitochondrial biogenesis in cultured macrophages exposed to asbestos and targeting redox signaling prevented the profibrotic polarization of lung macrophages by reducing the production of profibrotic molecules (He et al., 2019). The mechanism by which NOX4-induced mt ROS lead to epithelial cell death is yet to be investigated. NOX-induced mt ROS leading to mitochondrial biogenesis and profibrotic phenotype in macrophages has been demonstrated (He et al., 2019). Suppression of mitochondrial biogenesis and bioenergetics in lung fibroblasts by NOX4 *via* Nrf2 pathway is also reported (Bernard et al., 2017). NOX4-derived ROS is demonstrated to be a crucial down-stream mediator of TGF-β1-induced alveolar epithelial cell death. NOX4 deficiency and acute inhibition of NOX4 prevented TGF-β1-induced cell death in primary alveolar epithelial cells (Carnesecchi et al., 2011). It is likely that TGF-β1 is not the only factor contributing to NOX4 expression in the bleomycin model. Indeed, NOX4 upregulation may be a part of cellular stress responses, such as endoplasmic reticulum stress (Pedruzzi et al., 2004; Koli et al., 2008). As NOX4 is downstream of TGF-β, inhibition of NOX4 would be expected to be more effective than TGF-β1 inhibition in preventing the development of epithelial cell death and the subsequent injury in the lungs.

## Conclusion

**FIGURE 12:**
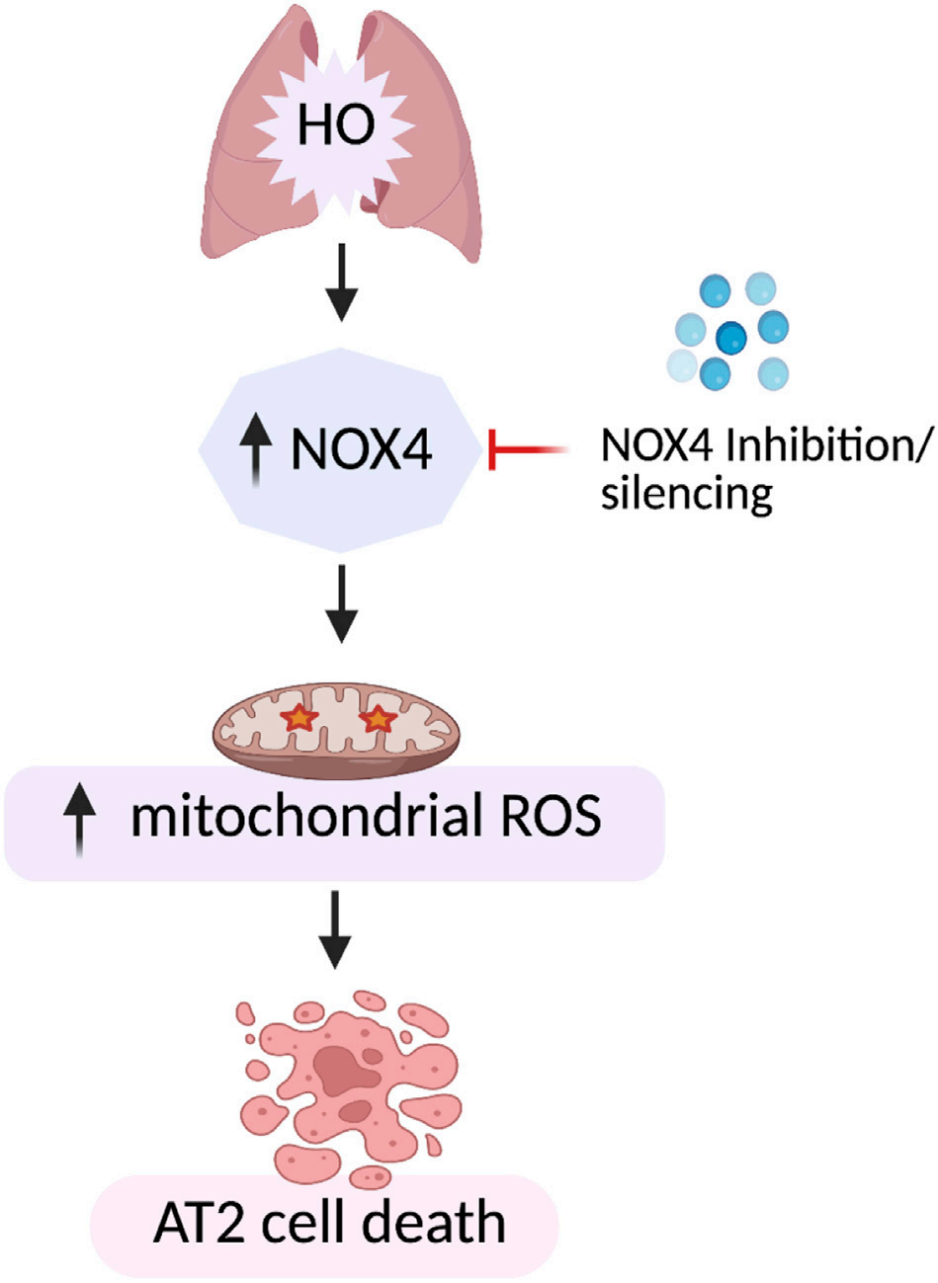
Model of HO-induced apoptosis of lung epithelial cells. HO causes an increase in NOX4 expression leading to increased mt ROS production resulting in lung epithelial cell apoptosis. This HO-induced mt ROS production and apoptosis was reduced by silencing NOX4 or mt ROS scavenging by MitoTEMPO.

Our data demonstrates increased expression of NOX4 in hyperoxic mouse lung tissues as well as mouse lung epithelial (MLE-12) cells suggesting a key role for NOX4 in HALI. Genetic deletion of *Nox4* in lung epithelium AT2 cells protected mice from HALI. A mechanistic link between NOX4 and mt ROS leading to epithelial cell death in lungs is established (Figure 12). These results suggest NOX4/ mt ROS as potential therapeutic targets for the treatment of HALI.

## Data Availability

The raw data supporting the conclusions of this article will be made available by the authors, without undue reservation.
